# Enhancing HCV NS3 Inhibitor Classification with Optimized Molecular Fingerprints Using Random Forest

**DOI:** 10.3390/ijms26062680

**Published:** 2025-03-17

**Authors:** Sema Atasever

**Affiliations:** Department of Computer Engineering, Faculty of Engineering and Architecture, Nevsehir Haci Bektas Veli University, 50300 Nevşehir, Turkey; sema@nevsehir.edu.tr

**Keywords:** machine learning, computational drug design, HCV NS3 inhibitors, QSAR, molecular descriptor optimization

## Abstract

The classification of Hepatitis C virus (HCV) NS3 inhibitors is essential for identifying potential antiviral agents through computational methods. This study aims to develop an optimized machine learning (ML) model using random forest (RF) and molecular fingerprints to accurately classify HCV NS3 inhibitors. A dataset of 965 molecules was retrieved from the ChEMBL database, and 290 bioactive compounds were selected for model training. Twelve molecular fingerprint descriptors were tested, and the CDK graph-only fingerprint yielded the best performance. In addition to RF, performance comparisons of other classifiers such as instance-based k-nearest neighbor (IBk), logistic regression (LR), AdaBoost, and OneR were conducted using WEKA with various molecular fingerprint descriptors. The optimized RF model achieved an accuracy of 89.6552%, a mean absolute error (MAE) of 0.2114, a root mean square error (RMSE) of 0.3304, and a Matthews correlation coefficient (MCC) of 0.7950 on the test set. These results highlight the effectiveness of optimized molecular fingerprints in enhancing virtual screening (VS) for HCV inhibitors. This approach offers a data-driven method for drug discovery.

## 1. Introduction

Globally, hepatitis C affects 50 million people chronically, with 1 million new cases annually [1], highlighting its recognition as a severe public health issue and driving ongoing clinical research into direct-acting antivirals to combat the infection [2]. Current medicinal chemistry theory and practice focus on possible therapeutic compounds’ physicochemical qualities, which determine attrition [3]. Understanding the impact of physicochemical qualities on the biological activity of compounds requires a thorough investigation of their chemical functions and biological activity [4]. Advances in machine learning (ML) and data analysis have made drug discovery processes more efficient by providing new research opportunities and treatment options in bioinformatics. Methods such as in silico drug discovery, using molecular simulation and artificial intelligence, help solve the problems of the high cost and low success rate of traditional methods [5]. Computational methods are essential in interdisciplinary research for discovering new drugs. To make a genuine difference in drug discovery, it is vital to understand the science underpinning computational tools, both the limits and the potential they provide [4,6]. QSAR is a computational modeling technique that identifies significant correlations between a molecule’s structure and its biological function. Originating over a century ago, QSAR has become an essential predictive tool in pharmaceuticals [7]. The identification or encoding of a chemical structure by means of molecular descriptors, therefore, is a critical point in QSAR. Numerous cheminformatics software packages have been developed, which allow one to calculate thousands of molecular descriptors [8].

Many VS methods have been created with varying molecular representations, speeds, and accuracy [9]. Comparative studies have shown that ligand-based methods, especially 2D fingerprints, are faster and more effective in terms of hit enrichment than 3D shape similarity and structure-based methods, making them the preferred choice in VS workflows [10]. The application of different molecular descriptors yields an in silico model with outstanding performance [11] Thus, the objective of this investigation is to examine the impact of molecular fingerprints on the forecasting of bioactivity of hepatitis C virus NS3 inhibitors. The advantageous functions of QSAR models have led to the utilization of numerous HCV inhibitors as datasets to establish predictive models that facilitate rational drug design. A collection of 290 inhibitors possessing established IC50 values against HCV NS3 was compiled for the purposes of this study. Predictive models were constructed using the RF algorithm. The resulting QSAR model was adept at accurately classifying compounds as either “active” or “inactive” against the HCV NS3, as demonstrated by the accuracy, MAE, RMSE, and MCC, which proved to be statistically proficient. Consequently, this knowledge has the potential to be employed in the development of more potent and specific drugs aimed at combating HCV. The contribution of this work is to enhance the hepatitis C Virus NS3 inhibitor classification using molecular fingerprint descriptors and ML models.

A well-balanced dataset with appropriate activity labels is crucial for the reliable classification of HCV NS3 inhibitors. The selection of computational tools, including ML algorithms and molecular fingerprints, is guided by nature and distribution of available data. In this study, the dataset was carefully curated to ensure the accurate assignment of bioactivity labels. This approach helps reduce potential biases in model training. A systematic evaluation of 12 molecular fingerprint descriptors was conducted to determine their effectiveness for classification. Through this analysis, the CDK graph-only fingerprint was identified as the most effective. This finding highlights the importance of selecting the right molecular representation. The optimized RF model achieves high accuracy and strong performance metrics. These results emphasize the importance of data quality and descriptor selection in enhancing predictive models for virtual screening. These findings show that using well-organized datasets and suitable computational methods is important for improving drug discovery.

### Literature Review

Considerable literature has been dedicated to exploring the current outlook of hepatitis C Virus NS3 inhibitors, encompassing various aspects such as the discovery of novel inhibitors and the understanding of NS3 protein function.

Musmuca et al. [12] demonstrated how a full computational method and biological research identified new molecular scaffolds for NS5B polymerase. Wang et al. [13] created classification models using the support vector machine (SVM) method. Structural analysis revealed a unique substructure such as cyclopropyl with the acylsulfonamide group found in active inhibitors. SVM models, especially Model 2B, which contains 11 descriptors representing basic structural information, showed strong classification ability. The SVM model (Model 2B) of this study may be valuable in VS to discover new HCV NS3 protease inhibitors by utilizing defined characteristic substructures. A research study [14], aimed at developing inhibitors for the HCV NS3/4A protease, a critical drug target in Hepatitis C virus, was conducted. These inhibitors exhibited effectiveness against both the wildtype and mutant forms of the protease, with compound 22 displaying the highest activity. Notably, these inhibitors demonstrated specificity towards the viral protease and showcased a therapeutic range in cell viability assays. In comparison, the approved drug simeprevir exhibited decreased potency against the mutant enzyme in comparison to the wildtype. The research conducted by Zhou et al. [15] revealed that HCV infection leads to a decrease in PPM1A levels in hepatoma cells through the action of NS3. Expression of PPM1A is notably reduced in tumor tissues of hepatocellular carcinoma (HCC). NS3 interacts with PPM1A, facilitating its degradation via ubiquitination. In their study, Iwai et al. [16] investigated the role of the NS3 protein. Through various experiments, the researchers demonstrated that NS3 interacts with the SRCAP and p400 proteins. Significantly, the ability of NS3 to activate the notch-mediated transcription of the Hes-1 promoter was markedly reduced when both SRCAP and p400 were silenced. Kamboj et al.’s study [17] discussed the development of an “Anti-HCV” platform that used ML. The models performed strongly on cross validation and independent validation datasets, and potential repurposed drugs were identified and further validated through molecular docking. These findings suggest that the identified drugs have the potential to be useful in the development of antiviral drugs against HCV. Hentabli et al.’s study [18] discussed the concept of molecular similarity in drug design based on the idea that structurally similar molecules will have similar properties. Graphic-based molecular descriptors demonstrated their superiority in identifying various datasets by comparing them with various standard descriptors in simulated virtual scan experiments and outperformed the previously proposed LWDOSM and Lingo-DOSM descriptors. Gong et al., [19] in their study for early prediction of nephrotoxicity, manually collected 777 valid drug data and created a classification model with different ML algorithms. 

Inspired by these studies that described and considered the current need, it is assumed that the classification of the biological activity of HCV NS3 inhibitors can be predicted using the most appropriate ML method and molecular fingerprint descriptors sets.

## 2. Results and Discussion

In this section, we present a detailed overview of the results obtained from our proposed model. We begin by describing the experimental setup and outlining the process of dataset collection and preparation. Following this, we provide a comprehensive analysis of the experimental results, highlighting the performance metrics and the effectiveness of the proposed approach.

### 2.1. Experimental Setup

The implementation was carried out using WEKA 3.8.6 on a Windows 10 Pro system equipped with an Intel i9 core processor. The system operates on a 64-bit architecture, featuring 128 GB of RAM and an NVIDIA GeForce RTX 3080 GPU. All experiments were conducted within the WEKA environment, ensuring consistency and reproducibility throughout the evaluation process.

### 2.2. Chemical Space Analysis

**Figure 1:**
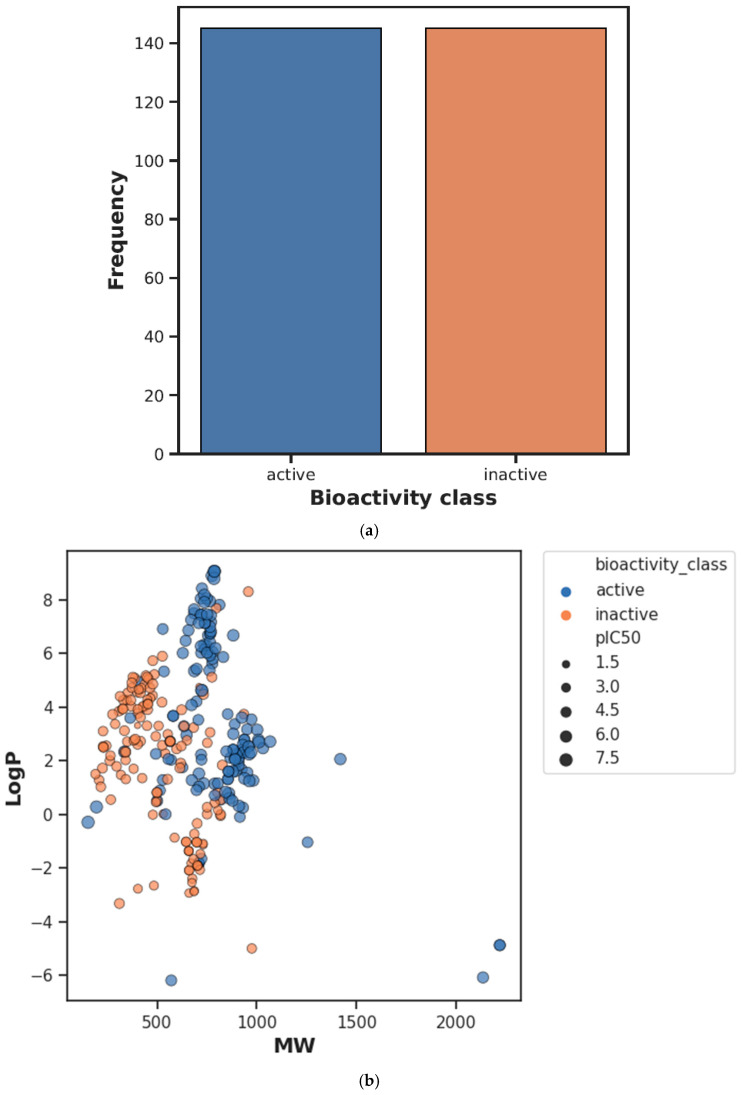
(**a**) Frequency plot of bioactivity classes. (**b**) Scatter plot of MW vs. LogP. Active and inactive compounds are shown in blue and orange colors, respectively.

**Figure 2:**
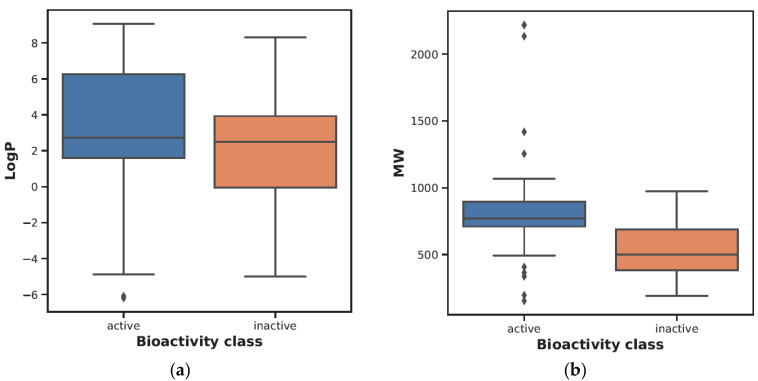

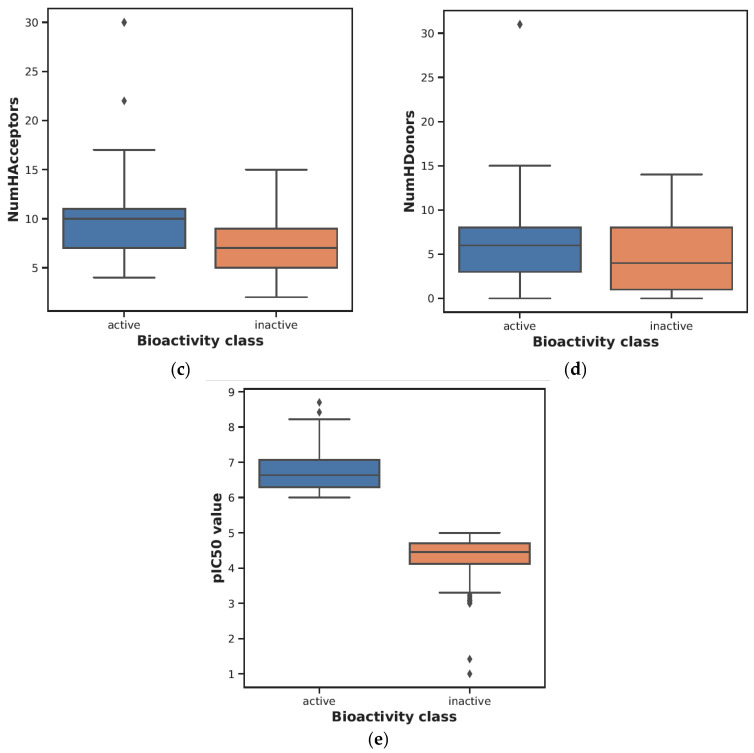
(**a**–**e**): A box plot illustrating the comparison of bioactivity classes between active and inactive compounds.

Performing chemical space analysis is a crucial step in investigating the differences between active and inactive compounds. In this study, it was aimed to obtain information about the general chemical space by visualizing the distribution of actives and inactives according to MW versus LogP. Then, using Ro5 descriptors, the two groups were compared. These properties are based on the observation that most drugs are relatively large lipophilic molecules containing MW, LogP, hydrogen bond donor number (nHBDon), and hydrogen bond acceptor number (nHBAcc). The visualization of MW as a function of LogP is presented in Figure 1b. In addition, the statistical analysis results showed a significant distinction between active and inactive compounds using the Mann–Whitney U test (see Table 1). In addition, the LogP, MW, nHBAcc, and nHBDon values of the active compounds were higher than the inactive compounds (see Figure 2).

### 2.3. QSAR Modeling and Bioactivity Class Analysis

In this study, interpretable molecular fingerprints were generated using the PaDEL-Descriptor 2.21 software. A comprehensive inventory of these fingerprints, accompanied by their respective explications, can be perused in Table 2. To undertake EDA, the Ro5 descriptor was engaged, which led to the identification of a total of 456 bioactivity data points. To discern significant differences between bioactivity classes, the Mann–Whitney U test was employed. A summary of the Mann–Whitney U test outcomes about the significant dissimilarities observed in both bioactivity classes is presented in Table 1.

As can be seen in Table 1, the Mann–Whitney U test was used to determine whether the examined bioactive molecules differed according to their bioactivity classes. The interpretation of the five descriptors LogP, MW, NumHAcceptors, NumHDonors, and pIC50 highlights that both classes are significantly different.

### 2.4. Model Evaluation

The effectiveness of a QSAR model’s estimation performance is contingent upon the composite descriptors as well as the employed estimator. In this study, RF was implemented due to its interpretability in various applications and the success of previous models [4]. The dataset used to train the model was divided into an 80/20 ratio between training and testing. The evaluation of the performance of the RF model was conducted based on the accuracy, MAE, RMSE, and MCC. The RF model attained an accuracy score of 0.896552 for the test data, utilizing solely the CDK graph only molecular descriptor (see Table 3).

Table 3 presents a comparison of five different classifier algorithms’ performance using various molecular fingerprint descriptors in WEKA. These classifiers include RF, IBk, LR, AdaBoost, and OneR. According to the results presented in Table 3, the RF algorithm using the CDK graph-only fingerprint descriptor achieved the best performance, with the highest accuracy and robustness across various evaluation metrics. Table 3 focuses on various metrics including accuracy, MAE, RMSE, and MCC across training, 10-fold cross-validation, and test datasets. The analysis indicates that the CDK graph-only fingerprint descriptor exhibits superior performance among the evaluated classes. It achieves the highest accuracy on the test dataset at 89.6552%. This descriptor shows the lowest MAE (0.2114) and RMSE (0.3304) and the highest MCC (0.7950), proving its effectiveness in molecular classification tasks. The results from both the 10-fold CV, with an accuracy of 88.9655%, and the test set validation, confirm that the CDK graph-only fingerprint descriptor outperforms other classes in accuracy and reliability in model predictions. This enhanced performance can be attributed to the unique structural representation captured by the CDK graph-only descriptor, which possibly encodes molecular features more effectively than other descriptors. The consistent results across various metrics and datasets show how well the CDK graph-only fingerprint uses the RF algorithm’s predictive power for classifying molecules. A well-balanced dataset with appropriate activity labels is crucial for the reliable classification of HCV NS3 inhibitors. The selection of computational tools, including ML algorithms and molecular fingerprints, is guided by nature and distribution of available data. In this study, the dataset was carefully curated to ensure the accurate assignment of bioactivity labels. This approach helped to reduce potential biases in model training. A systematic evaluation of 12 molecular fingerprint descriptors was conducted to determine their effectiveness for classification. Through this analysis, the CDK graph-only fingerprint was identified as the most effective. This finding highlights the importance of selecting the right molecular representation. The optimized RF model achieved high accuracy and strong performance metrics. These results emphasize the importance of data quality and descriptor selection in enhancing predictive models for virtual screening. These findings show that using well-organized datasets and suitable computational methods is important for improving drug discovery.

An essential aspect of this study was the evaluation of molecular descriptors and their impact on model performance. Among the 12 molecular fingerprints tested, the CDK graph-only fingerprint demonstrated the highest predictive ability. This finding indicates that structural connectivity plays a key role in distinguishing active and inactive HCV NS3 inhibitors. The strong classification performance of the RF model, with an accuracy of 89.6552% and an MCC of 0.7950, highlights its effectiveness. This result underscores the relevance of descriptor selection in enhancing predictive capabilities. Similarly, the study by Phanus-Umporn et al. [27] demonstrated that substructure fingerprints, combined with the RF method, achieved the best performance. Moreover, this approach provides an interpretable set of descriptors, reinforcing the importance of feature selection in predictive modeling. The findings suggest that molecular features related to graph-based representations are particularly informative for this classification task.

## 3. Materials and Methods

### 3.1. Dataset

The dataset used in this study was retrieved from the ChEMBL database (version 33) [28]. It specifically targets HCV NS3 (Target ChEMBL ID: CHEMBL1293269). The original dataset contained 1121 bioactivity data points from 965 compounds. To ensure data consistency, we applied a filtering process that retained only entries with standard type = ‘IC50’ and non-null standard value, resulting in 456 bioactivity data points.

To classify compounds, we categorized them based on their IC50 values:

Active: IC50 ≤ 1 μM;

Inactive: IC50 ≥ 10 μM;

Intermediate values (1–10 μM) were excluded.

**Figure 3:**
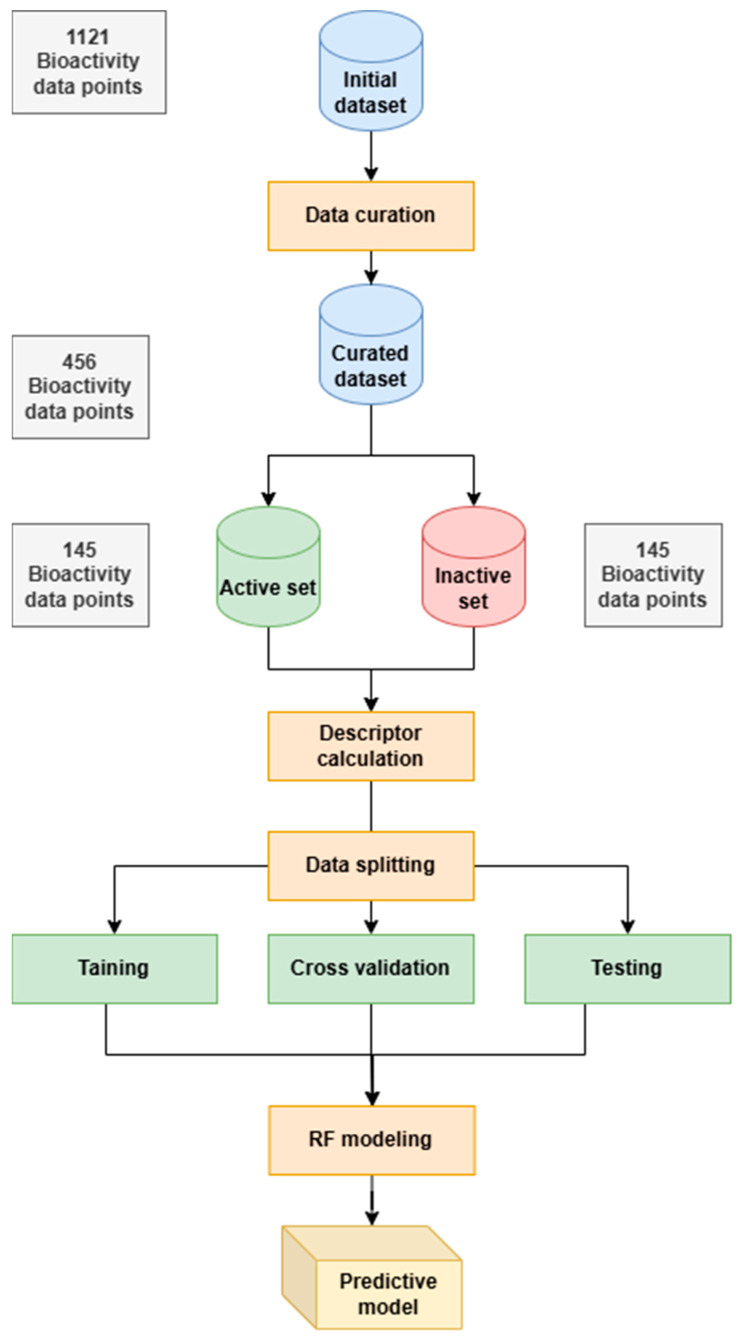
A schematic overview of the workflow for QSAR modeling.

This resulted in a curated dataset of 290 inhibitors, which was used for further analysis. The chemical space distributions of the dataset were evaluated using PaDEL-Descriptor 2.21 software [29], which calculates 12 groups of molecular descriptors and converts SMILES notation into molecular descriptors (see Figure 3).

### 3.2. Data Preprocessing

For improved model performance, the IC50 values were transformed into pIC50 values using the following equation:

pIC50 = −log_10_(IC50 × 10^−9^). (1)

Bioactive compounds were then labeled as active (≤1000 nM), inactive (≥10,000 nM), or intermediate classes (between 1000 and 10,000) according to their IC50 values [30].

**Figure 4:**
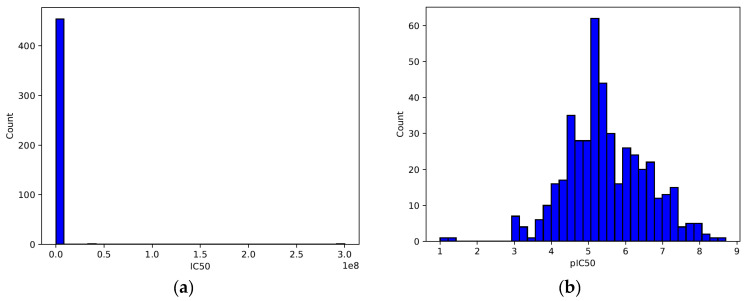
(**a**,**b**): Distribution of IC50 and pIC50 values.

The left plot (Figure 4a) shows the raw IC50 values in nanomoles (nM). The distribution appears highly skewed, with many values concentrated near the lower end and a few extreme values extending toward the right. This right-skewed distribution suggests that the dataset contains a wide range of IC50 values, with many compounds having low IC50 values but some compounds exhibiting extremely high IC50 values. Such skewness can create problems in modeling, as large-scale differences in IC50 values can dominate the learning process.

The right plot (Figure 4b) shows the distribution of the pIC50 values, which were obtained using Equation (1). Unlike the IC50 distribution, the pIC50 values follow a more normal-like (Gaussian) distribution, centered around 5–6 pIC50. The transformation compresses the large IC50 values, reducing the skewness and improving the numerical stability for ML models. This transformation is commonly used in cheminformatics and QSAR modeling, as it better represents biological activity on a logarithmic scale.

The decision to use classification instead of a regression approach was primarily guided by the characteristics of the data. Additionally, the intended application of the model played a crucial role in this choice. As shown in Figure 4b, the pIC50 values exhibit a near-Gaussian distribution, which could be suitable for regression. However, the original IC50 values (Figure 4a) were highly skewed, spanning several orders of magnitude. This skewness can introduce challenges in regression modeling. The transformation to pIC50 helped reduce the skewness. However, classification was ultimately chosen due to its practical advantages in drug discovery, where compounds are often categorized based on predefined activity thresholds. This approach aligns with common cheminformatics practices [31]. It also enhances interpretability by allowing the direct identification of active, intermediate, and inactive compounds. Nevertheless, future work could explore regression-based modeling to capture finer variations in activity levels.

### 3.3. Molecular Fingerprint

Various molecular descriptors capture different aspects of molecules and are classified according to their size: 1D descriptors for bulk properties and physicochemical parameters, 2D descriptors for structural fragments, and 3D descriptors for molecular shape [32]. Molecular fingerprints, typically depicted as a string of numbers 1 and 0 with a fixed length, define the characteristics of a molecule through a binary string of structural information. In this representation, the number 1 signifies the presence of a substructure, while the number 0 indicates its absence [33].

Molecular descriptors capture various structural and physicochemical properties of compounds. They are classified as [32]:

One-dimensional descriptors: Bulk properties and physicochemical parameters.

Two-dimensional descriptors: Structural fragments.

Three-dimensional descriptors: Molecular shape.

Molecular fingerprints, represented as binary strings, encode molecular structure where 1 indicates the presence of a substructure, and 0 indicates its absence [33].

In this study, we used 12 distinct fingerprints, computed using PaDEL-Descriptor 2.21 software [29], as follows:

CDK fingerprint (1024 bits);

CDK extended fingerprint (1024 bits);

Estate fingerprint (79 bits);

CDK graph-only fingerprint (1024 bits);

Molecular access system (MACCS) (166 bits);

PubChem fingerprint (881 bits)l

Substructure fingerprint (307 bits);

Substructure count fingerprint (307 patterns);

Klekota–Roth fingerprint (4860 bits);

Klekota–Roth count (4860 Roth count);

Two-dimensional atom pairs fingerprint (780 bits);

Two-dimensional atom pairs count (780 2D atom pairs count)

A summary of all the fingerprints and their feature counts is provided in Table 2 [27].

To remove redundant descriptors, WEKA’s ‘RemoveUseless’ filter was applied, eliminating descriptors that did not vary across instances. The processed dataset, along with the computed attributes, is available as Supplemental Files S1–S12.

### 3.4. QSAR Modeling

Quantitative structure–activity relationship (QSAR) modeling was performed using WEKA 3.8.6, applying 10-fold cross validation on data split into an 80/20 ratio for training and testing.

The model performance was evaluated using four key metrics:

Accuracy,

Mean absolute error (MAE),

Root mean square error (RMSE),

Matthews correlation coefficient (MCC).

Different molecular fingerprint descriptors were tested to determine their predictive power for bioactivity classification.

### 3.5. Exploratory Data Analysis (EDA)

**Figure 5:**
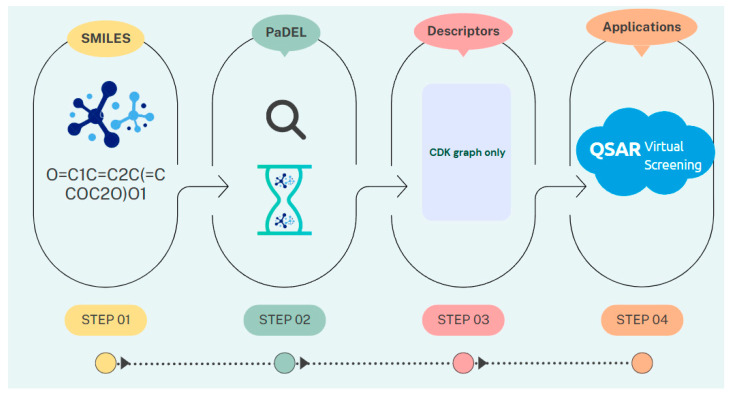
Main steps of EDA.

The study presents a schematic summary of the EDA modeling workflow, as depicted in Figure 5. Additionally, Figure 1a illustrates the frequency graph representing the active and inactive classes, while Figure 1b displays a scatterplot comparing the molecular weight (MW) with LogP.

Data analysis and ML have become essential components of contemporary scientific methodology, providing automated methods for predicting additional information based on observations. One prevalent technique for classification and regression is the RF method [34,35]. In the RF approach, the classification process initiates at the root node, where the dataset splits, based on chosen descriptors, to ensure that distinct activities are primarily assigned to different branches. The final classification is determined by aggregating the outcomes of all trees through a majority vote [4]. 

RF models are chosen as baseline models due to their widespread use and effectiveness in predicting biological activity in various studies [36], often outperforming other ML methods according to recent benchmarking studies [37]. 

**Figure 6:**
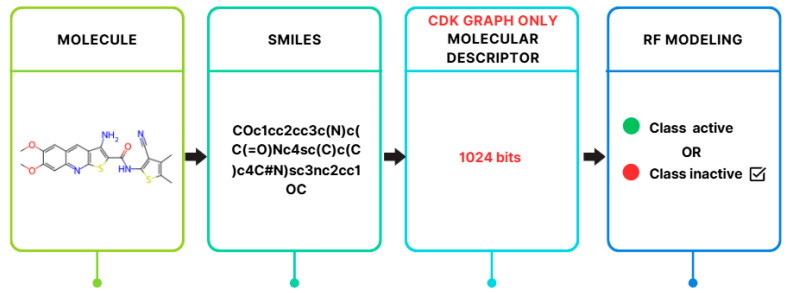
Overview of the RF method.

In this study, the RF model is focused on due to its widespread use and strong performance in drug discovery applications. According to Atasever’s study [5], RF is identified as the most used ML method in this domain. It appears in 53% of the studies examined (63 studies). This widespread adoption is considered evidence of its reliability and effectiveness in predicting molecular properties and biological activities. RF is particularly well-suited for cheminformatics. It is found to outperform other ML models in handling complex, noisy, and high-dimensional datasets [38]. One of RF’s key strengths is its robustness to overfitting, as it maintains a balance between bias and variance. This balance allows the model to generalize better to unseen data. RF also offers feature importance analysis, helping researchers identify the most relevant molecular descriptors. As a result, RF is regarded as both a powerful predictive tool and an interpretable one. Its successful application in past drug discovery studies makes it a reliable and widely accepted model in the field. Given RF’s dominance in literature and its superior performance in this study (as shown in Table 3), it was prioritized for deeper analysis. Therefore, in this study, the RF classifier was applied using the WEKA tool. An overview of the modeling method used in this study is presented in Figure 6.

### 3.6. Assessment of Model Performance

The assessment of the model performance was carried out using four metrics, namely accuracy, MAE, RMSE, and MCC.

The evaluation of each model’s quality considered parameters such as true positives (TP), false positives (FP), true negatives (TN), and false negatives (FN). The model’s performance was assessed using various statistical metrics, including comprehensive classification accuracy (Acc), MAE, RMSE, and MCC, to determine its proficiency [4].

(2) $$\mathrm{Acc}=\frac{TP-TN}{\left(TP+TN+FP+FN\right)}*100$$

The MAE quantifies the average degree of errors in a set of projections, representing the average difference between the predicted and actual data with equal weight assigned to each individual variance. The formula for calculating MAE is as follows [10]:

(3) $$\mathrm{MAE}=\frac{1}{n}\sum_{j=1}^{n}\left|y_{i}-{\hat{y}}_{i}\right|.$$

The MAE compares the actual output (yᵢ) with the model’s prediction (ŷᵢ) and represents the average square error of the estimates. By calculating the squared difference between the estimates and the target, and then averaging those values, MAE provides insights into the model’s performance. A higher MAE value indicates poorer model performance, as it signifies larger errors. The MAE value must be greater than zero, as the process involves squaring the individual prediction-wise errors before summing them. Ideally, a perfect model would have an MAE value close to zero [39].

The RMSE is a mathematical rule used to assess the average magnitude of an error, representing the square root of the average square difference between the predictions and actual observations [35,39].

(4) $$\mathrm{RMSE}=\sqrt{\frac{1}{n}}\sum_{j=1}^{n}{\left(y_{i}-{\hat{y}}_{i}\right)}^{2}$$

The MAE and RMSE are metrics used to quantify the average errors of a model in the units of the variables. Both metrics can range from 0 to ∞ and do not consider the direction of the error. In cases where negative outputs indicate better performance, lower values are desirable. The RMSE, obtained by taking the square root of the mean square error, holds significance due to its handling of larger errors. By squaring the errors before averaging, the RMSE assigns greater importance to significant errors. Therefore, the RMSE proves more informative when large errors have more unfavorable consequences [4,37].

(5) $$\mathrm{MCC}=\frac{TP*TN-FP*FN}{\sqrt{\left(TP+FP\right)\left(TP+FN\right)\left(TN+FP\right)\left(TN+FN\right)}}$$

The symbols TP, TN, FP, and FN correspond to true positives, true negatives, false positives, and false negatives, respectively, representing different instances within the context.

## 4. Conclusions

Innovative anti-HCV drugs are needed to combat the rising worldwide prevalence of HCV infections, and molecular descriptors are crucial to model performance. In this research, a total of 965 compounds were compiled. Using the RF method and different molecular fingerprints, 12 models were created to classify 290 bioactivity data points. The RF model with CDK graph-only fingerprints demonstrated the best classification ability, achieving accuracy, MAE, RMSE, and MCC of 89.6552%, 0.2114, 0.3304, and 0.7950 on the test set, respectively. The comparison of several classifiers such as RF, IBk, LR, AdaBoost, and OneR highlights the adaptability of using different molecular fingerprint descriptors in WEKA for this study. The best RF model presented in this study can be used as a general guide for the data-driven design of potentially active HCV NS3 protease inhibitors in virtual screening.

## Figures and Tables

**Table 1:** The results of the Mann–Whitney U test pertaining to the bioactivity classes of the investigated bioactive molecules.

Descriptor	Statistics	*p*	Alpha	Interpretation
LogP	13,400.5	0.000053	0.05	Different distribution (reject H0)
MW	17,897.5	4.550260 × 10^−25^	0.05	Different distribution (reject H0)
NumHAcceptors	14,950.5	3.587758 × 10^−10^	0.05	Different distribution (reject H0)
NumHDonors	13,257.0	0.000111	0.05	Different distribution (reject H0)
pIC50	21,025.0	4.634027 × 10^−49^	0.05	Different distribution (reject H0)

**Table 2:** Molecular descriptors list.

Fingerprint	#Features	Description	References
CDK	1024	Fingerprint of length 1024 and search depth of 8	[20]
CDK extended	1024	Extends the fingerprint with additional bits describing ring features	[20]
CDK graph only	1024	A special version that considers only the connectivity and not bond order	[20]
E-state	79	Electrotopological state atom types	[21]
MACCS	166	Binary representation of chemical features defined by MACCS keys	[22]
PubChem	881	Binary representation of substructures defined by PubChem	[23]
Substructure	307	Presence of SMARTS patterns for functional groups	[24]
Substructure count	307	Count of SMARTS patterns for functional groups	[24]
Klekota–Roth	4860	Presence of chemical substructures	[25]
Klekota–Roth count	4860	Count of chemical substructures	[25]
2D atom pairs	780	Presence of atom pairs at various topological distances	[26]
2D atom pairs count	780	Count of atom pairs at various topological distances	[26]

**Table 3:** Comparative performance of different ML models on various molecular descriptor classes.

			Training Set				Ten-Fold CV Set				Test Set			
Descriptor Class	Method	A *	Acc_Train_ (%)	MAE	RMSE	MCC	Acc_Cv_ (%)	MAE	RMSE	MCC	Acc_Test_ (%)	MAE	RMSE	MCC
CDK	RF	1022	97.9310	0.0870	0.1469	0.9790	88.2759	0.2201	0.3232	0.7660	86.2069	0.2263	0.3490	0.7320
CDK	IBk	1022	97.9310	0.0254	0.1059	0.9590	86.2069	0.1461	0.3711	0.7240	86.2069	0.1466	0.3726	0.7320
CDK	LR	1022	97.9310	0.0224	0.1059	0.9590	74.4828	0.2558	0.4983	0.4930	79.3103	0.2094	0.4460	0.6130
CDK	AdaBoost	1022	88.6207	0.1805	0.2950	0.7750	82.4138	0.2170	0.3584	0.6510	86.2069	0.1974	0.3451	0.7320
CDK	OneR	1022	78.2759	0.2172	0.4661	0.5720	74.4828	0.2552	0.5051	0.4950	70.6897	0.2931	0.5414	0.4410
CDK extended	RF	1007	97.9310	0.0845	0.1440	0.9590	87.5862	0.2172	0.3238	0.7520	86.2069	0.2170	0.3456	0.7260
CDK extended	IBk	1007	97.931	0.0254	0.1059	0.9590	86.5517	0.1427	0.3665	0.7310	86.2069	0.1466	0.3726	0.7320
CDK extended	LR	1007	97.9310	0.0224	0.1059	0.9590	75.1724	0.2506	0.4935	0.5060	77.5862	0.2330	0.4700	0.5560
CDK extended	OneR	1007	75.8621	0.2414	0.4913	0.5260	73.1034	0.2690	0.5186	0.4660	63.7931	0.3621	0.6017	0.2890
CDK extended	AdaBoost	1007	88.2759	0.1857	0.2896	0.7660	80.0000	0.2402	0.3756	0.6020	75.8621	0.2706	0.4054	0.5730
Estate	RF	34	94.8276	0.1095	0.2004	0.8980	87.9310	0.1996	0.3223	0.7600	82.7586	0.2366	0.3781	0.6630
Estate	IBk	34	94.8276	0.0697	0.1842	0.8990	87.2414	0.1555	0.3345	0.7480	82.7586	0.2127	0.4128	0.6720
Estate	LR	34	88.6207	0.1620	0.2856	0.7730	80.3448	0.2293	0.4017	0.6110	75.8621	0.2515	0.4364	0.5420
Estate	AdaBoost	34	81.0345	0.3266	0.3827	0.6320	78.6207	0.3253	0.3926	0.5790	79.3103	0.3309	0.3997	0.6280
Estate	OneR	34	69.6552	0.3034	0.5509	0.4110	67.2414	0.3276	0.5724	0.3500	63.7931	0.3621	0.6017	0.4140
CDK graph only	RF	979	97.5862	0.0864	0.1582	0.9520	88.9655	0.1994	0.3181	0.7790	89.6552	0.2114	0.3304	0.7950
CDK graph only	IBk	979	97.5862	0.0346	0.1264	0.9520	86.2069	0.1541	0.3694	0.7240	84.4828	0.1661	0.3659	0.7020
CDK graph only	LR	979	97.5862	0.0320	0.1264	0.9520	73.7931	0.2690	0.5040	0.4790	74.1379	0.2383	0.4695	0.4830
CDK graph only	AdaBoost	979	89.3103	0.2038	0.2959	0.7880	83.7931	0.2540	0.3558	0.6760	86.2069	0.1955	0.3094	0.7260
CDK graph only	OneR	979	72.7586	0.2724	0.5219	0.4610	66.5517	0.3345	0.5783	0.3350	67.2414	0.3276	0.5724	0.3800
MACCS	RF	145	98.2759	0.0769	0.1393	0.9660	87.2414	0.1946	0.3092	0.7450	87.9310	0.1990	0.3104	0.7630
MACCS	IBk	145	98.2759	0.0229	0.1003	0.9660	86.8966	0.1383	0.3585	0.7390	86.2069	0.1373	0.3548	0.7260
MACCS	LR	145	97.2414	0.0329	0.1286	0.9450	74.1379	0.2619	0.5026	0.4830	82.7586	0.1880	0.4218	0.6630
MACCS	AdaBoost	145	83.7931	0.2241	0.3337	0.6810	82.7586	0.2456	0.3600	0.6570	84.4828	0.2356	0.3568	0.7020
MACCS	OneR	145	75.1724	0.2483	0.4983	0.5060	75.1724	0.2483	0.4983	0.5060	70.6897	0.2931	0.5414	0.4130
PubChem	RF	561	98.2759	0.0806	0.1429	0.9660	87.2414	0.1927	0.3101	0.7450	87.9310	0.2118	0.3467	0.7710
PubChem	IBk	561	98.2759	0.0229	0.1003	0.9660	84.8276	0.1527	0.3738	0.6980	87.9310	0.1603	0.3695	0.7710
PubChem	LR	561	98.2759	0.0201	0.1003	0.9660	71.7241	0.2849	0.5273	0.4380	84.4828	0.1568	0.3811	0.7020
PubChem	AdaBoost	561	85.8621	0.1838	0.3109	0.7180	86.2069	0.1932	0.3308	0.7240	86.2069	0.1863	0.3436	0.7420
PubChem	OneR	561	85.8621	0.1414	0.3760	0.7180	85.8621	0.1414	0.3760	0.7180	84.4828	0.1552	0.3939	0.7130
Substructure	RF	101	94.1379	0.1102	0.2039	0.8850	86.8966	0.1971	0.3188	0.7380	82.7586	0.2200	0.3383	0.6570
Substructure	IBk	101	94.1379	0.0744	0.1901	0.8850	86.2069	0.1590	0.3434	0.7260	81.0345	0.2063	0.4109	0.6320
Substructure	LR	101	94.1379	0.0735	0.1916	0.8840	78.2759	0.2127	0.4242	0.5660	81.0345	0.1938	0.4169	0.6210
Substructure	AdaBoost	101	84.1379	0.2782	0.3489	0.6940	79.3103	0.2974	0.3793	0.5970	67.2414	0.3210	0.4155	0.4130
Substructure	OneR	101	70.6897	0.2931	0.5414	0.4990	68.2759	0.3172	0.5632	0.4140	72.4138	0.2759	0.5252	0.5030
Substructure count	RF	105	98.2759	0.0699	0.1355	0.9660	89.3103	0.1791	0.3072	0.7870	87.9310	0.1848	0.3112	0.7710
Substructure count	IBk	105	98.2759	0.0219	0.0974	0.9660	85.1724	0.1592	0.3885	0.7030	82.7586	0.1721	0.4001	0.6630
Substructure count	LR	105	97.5862	0.0307	0.1237	0.9520	78.2759	0.2250	0.4655	0.5670	82.7586	0.1799	0.4177	0.6550
Substructure count	AdaBoost	105	83.4483	0.2148	0.3275	0.6710	81.7241	0.2224	0.3559	0.6350	82.7586	0.2270	0.3629	0.6720
Substructure count	OneR	105	83.4483	0.1655	0.4068	0.6690	81.0345	0.1897	0.4355	0.6220	81.0345	0.1897	0.4355	0.6420
Klekota–Roth	RF	1094	98.6207	0.0763	0.1341	0.9730	87.2414	0.1966	0.3180	0.7450	86.2069	0.1782	0.3026	0.7420
Klekota–Roth	IBk	1094	98.6207	0.0186	0.0881	0.9730	86.8966	0.1402	0.3600	0.7380	84.4828	0.1486	0.3551	0.7020
Klekota–Roth	LR	1094	98.6207	0.0155	0.0881	0.9720	71.7241	0.2803	0.5205	0.4370	79.3103	0.2098	0.4438	0.6130
Klekota–Roth	AdaBoost	1094	86.2069	0.1946	0.3082	0.7330	86.5517	0.2017	0.3186	0.7310	87.9310	0.1894	0.3274	0.7710
Klekota–Roth	OneR	1094	78.2759	0.2172	0.4661	0.5730	74.8276	0.2517	0.5017	0.5030	77.5862	0.2241	0.4734	0.5510
Klekota–Roth count	RF	1097	98.6207	0.0730	0.1330	0.9730	87.2414	0.1874	0.3168	0.7450	84.4828	0.1868	0.3144	0.7020
Klekota–Roth count	IBk	1097	98.6207	0.0186	0.0881	0.9730	81.3793	0.1907	0.4268	0.6300	84.4828	0.1635	0.3947	0.7130
Klekota–Roth count	LR	1097	98.6207	0.0155	0.0881	0.9720	78.2759	0.2213	0.4632	0.5680	81.0345	0.1891	0.4124	0.6420
Klekota–Roth count	AdaBoost	1097	86.8966	0.1872	0.3072	0.7390	84.1379	0.2188	0.3557	0.6840	84.4828	0.2100	0.3430	0.7020
Klekota–Roth count	OneR	1097	83.1034	0.1690	0.4111	0.6670	76.5517	0.2345	0.4842	0.5310	72.4138	0.2759	0.5252	0.4480
2D atom pairs	RF	298	95.8621	0.1053	0.1920	0.9170	87.9310	0.2053	0.3174	0.7590	86.2069	0.2278	0.3290	0.7240
2D atom pairs	IBk	298	95.8621	0.0603	0.1707	0.9180	87.5862	0.1533	0.3405	0.7520	87.9310	0.1821	0.3584	0.7590
2D atom pairs	LR	298	94.8276	0.0670	0.1837	0.8970	76.2069	0.2579	0.4781	0.5260	79.3103	0.2533	0.4565	0.5860
2D atom pairs	AdaBoost	298	84.1379	0.2434	0.3322	0.6960	82.7586	0.2636	0.3609	0.6660	82.7586	0.3157	0.3990	0.6630
2D atom pairs	OneR	298	74.1379	0.2586	0.5085	0.5050	74.1379	0.2586	0.5085	0.5050	77.5862	0.2241	0.4734	0.5530
2D atom pairs count	RF	301	98.6207	0.0709	0.1318	0.9730	87.2414	0.1720	0.3025	0.7450	87.9310	0.1842	0.3265	0.7710
2D atom pairs count	IBk	301	98.6207	0.0186	0.0881	0.9730	84.8276	0.1548	0.3836	0.6970	84.4828	0.1636	0.3947	0.6900
2D atom pairs count	LR	301	98.6207	0.0155	0.0881	0.9730	73.4483	0.2634	0.5072	0.4700	84.4828	0.1567	0.3861	0.6890
2D atom pairs count	AdaBoost	301	86.5517	0.1729	0.2977	0.7320	84.8276	0.1978	0.3420	0.6980	81.0345	0.2026	0.3752	0.6320
2D atom pairs count	OneR	301	86.5517	0.1345	0.3667	0.7330	81.0345	0.1897	0.4355	0.6210	82.7586	0.1724	0.4152	0.6720

A *: denotes the number of attributes remaining after applying WEKA’s ‘RemoveUseless’ filter.

## Data Availability

The data used in this study are available in the Supplementary section.

## Supplementary Materials

The following data can be downloaded at https://www.mdpi.com/article/10.3390/ijms26062680/s1.
